# Evaluation of Erectile Dysfunction as a Consequence of Urethroplasty: A Prospective Observational Study

**DOI:** 10.7759/cureus.84936

**Published:** 2025-05-27

**Authors:** Devpriya Mitra, Vikas K Bansal, Deepak Sharma

**Affiliations:** 1 Urology, Robotics and Renal Transplant, Marengo Asia Hospitals, Faridabad, IND; 2 Urology, Yashoda Hospital and Research Centre, Ghaziabad, IND; 3 Urology, Shalby Sanar International Hospital, Gurgaon, IND

**Keywords:** erectile dysfunction, pelvic fracture urethral injury, urethral stricture complications, urethral stricture (us), urethroplasty, international index of erectile function

## Abstract

Objective

The objective of this study is to evaluate the change in erectile function in patients undergoing urethroplasty for anterior urethral strictures and pelvic fracture urethral injuries (PFUI) and to assess vascular insufficiency as the cause of erectile dysfunction after urethroplasty.

Material & Methods

For this study, 58 participants were enrolled. Eight patients had progressive perineal urethroplasty (PPU) for pelvic fracture urethral injury (PFUI), while five patients had urethroplasty for anterior urethral stricture (AUS). International Index of Erectile Function (IIEF-5) preoperative and postoperative scores were examined at three, six, nine, and 12 months. For every patient having an IIEF-5 score below 22, pharmacological color Doppler ultrasonography (CDUS) of penis was performed both before and after surgery at six months.

Results

Thirty men reported some degree of preoperative erectile dysfunction among patients who suffered from AUS. Postoperatively, at three months, 33 men reported erectile dysfunction, while after 12 months, 28 men reported erectile dysfunction. Additionally, there wasn’t any discernible change in IIEF ratings after a 12-month follow-up period (p-value 1.000). After three months, the mean IIEF score among PFUI patients significantly decreased to 16.75 ± 2.82 (p-value 0.002), but after twelve months, the IIEF score did not significantly alter. There was no discernible change in blood flow on postoperative colour Doppler ultrasonography.

Conclusions

Urethroplasty negatively alters erectile function during the early postoperative period. Still, after six to 12 months, most patients recover their erectile function to preoperative status. De novo erectile dysfunction is very rare post-urethroplasty. Following these operations, there aren't many vascular events that could cause erectile dysfunction.

## Introduction

Urethral stricture, a condition that results in scarring of the surrounding spongy tissues of the corpus spongiosum and urethral mucosa, causes the urethral lumen to shrink. Since the corpus spongiosum does not cover the neck of the bladder or prostatic urethra, this term "urethral stricture" refers to anterior urethral illness. Straddle injuries and blunt abdominal trauma are typically linked to pelvic fractures and posterior urethral injury.

Improved voiding function is the main objective of urethroplasty. Metrics like urine rate of flow and the lower urinary tract symptoms (LUTS) score have traditionally been used to assess postoperative outcomes following urethroplasty. The focus is increasingly shifting to other unintentional sexual dysfunctions like erectile dysfunction (ED) [1], penile curvature or chordee, ejaculatory dysfunction, as well as genital sensitivity problems, due to the great long-term success rates of urethroplasty, which addresses the voiding functions following surgery. Recent works of literature demonstrate that sexual dysfunction after urethroplasty is uncommon, about one percent (0-38 percent) after anterior urethroplasty and about three percent (0-34 percent) after pelvic fracture-related urethral injury repair [2].

Both pelvic fracture urethral injury (PFUI) and anterior urethral stricture (AUS) have been associated with ED [1,3]. The link between urethroplasty and erectile function remains controversial, despite the increased attention given to numerous andrological aspects of urethral stricture operations. A person's quality of life may suffer as a result of ED. It is typically assessed using validated questionnaires such as the International Index of Erectile Function (IIEF) score [1], which is an open-access questionnaire and has been previously employed by Erickson et al. in their prospective analysis of erectile dysfunction following anterior urethroplasty. To evaluate the vascular reasons for ED, color Doppler ultrasonography (CDUS) measures the penile blood flow's Resistive Index (RI), Peak Systolic Velocity (PSV), and end-diastolic velocity (EDV).

After anterior stricture urethroplasty and progressive perineal urethroplasty (PPU), we want to assess the prevalence of both pre-existing and de novo ED and rule out vasculogenic etiology as the reason for ED after urethroplasty at our institution.

## Materials and methods

From May 2018 to November 2019, we conducted a prospective, observational, hospital-based study on patients undergoing urethroplasty in the Department of Urology and Renal Transplantation. Following a meeting at BLK Superspeciality Hospital in New Delhi on June 21, 2018, the Scientific Research Committee (SRC) and the Institutional Ethics Committee (IEC) approved this study. Reference number for ethics committee approval: BLK/AARCE/JANUARY/2021/37.

Inclusion criteria

Males over 18 who are sexually active and have PFUI and anterior stricture of the urethra and who consented to intervention and follow-up must have a single-stage urethroplasty.

Exclusion criteria

Unable to comprehend the IIEF-5 score (Appendix 1), sexually inactive individuals, and patients in need of phased urethroplasty.

Informed written consents were taken from all patients, following which they underwent evaluation, including detailed clinical history, complete physical examination, and routine and specific investigations for urethral stricture. Specific investigations included: retrograde urethrogram (RGU), voiding cystourethrogram (VCUG), uroflowmetry with post-void residual urine, IIEF-5 score (Appendix 1), and pharmacological CDUS (for all patients having a preoperative IIEF-5 score of <22). The IIEF-5 questionnaire was given to all the patients, and the IIEF-5 score was recorded for the study. Patients having an IIEF-5 score of >22 were regarded as normal, 17 to 21 were considered as having mild ED, while a score of 12 to 16 was regarded as having mild to moderate ED, rate of eight to 11 was considered as having moderate ED, and score of <7 as severe ED [4]. Study participants were admitted to the cohort if they were willing and met the requirements for inclusion and exclusion. Every patient who had symptoms of epididymo-orchitis or urinary tract infections had suprapubic catheterization and antibiotic treatment before urethroplasty. Pharmacological CDUS of the penis was performed preoperatively on patients with ED who had an IIEF-5 score of less than 22 (Appendix 1).

Technique of pharmacological CDUS

A pharmacological CDUS flow study of a flaccid penis was performed on the patients in a supine position after they gave their consent and were informed of the treatment and its possible side effects. CDUS was done using high-frequency linear array transducers operating at 7.5 MHz. After injecting 30 mg of injectable papaverine into the corpora cavernosa, the PSV, EDV, and RI of the cavernosal artery were measured at the point where the proximal third and distal 2/3rd of the penile shaft intersected. Patients were left alone in a room for three to five minutes, and if needed, they were provided with visually stimulating media. Once again, PSV, EDV, and RI were observed serially. Venous insufficiency was described as EDV>5 cm/s, and arterial blockage as PSV <25 cm/s.

Technique of anterior urethroplasty

The stricture's length and position were taken into consideration when choosing the surgical method. RGU, VCUG films, and stricture length measurements were used to assess the urethral defect length before surgery. Using a 6Fr ureteroscope, the stricture's caliber was evaluated intraoperatively. Patients with short-segment (less than 2 cm) traumatic strictures in the bulbar urethra underwent end-to-end anastomotic urethroplasty (EEAU). Buccal mucosal graft (BMG) was utilized in substitution urethroplasty for penile urethral strictures, longer-segment bulbar urethral strictures, and inflammatory strictures. With the individual in the dorsal lithotomy position, a midline perineal incision was made, and after dissecting the urethra, the diseased urethral segment was identified. Before harvesting the BMG, the inner surface of the cheek was prepared, taking care to avoid Stensen's duct opening (parotid duct), which is situated across from the crown of the upper second molar tooth. Depending on the type of stricture and preference of the surgeon, graft urethroplasty was completed with positioning of graft dorsal onlay/dorsolaterally/dorsal inlay/double face (dorsal inlay+ventral onlay). In case of penile strictures, dorsal inlay/ dorsolateral urethroplasty has been done.

Bulbar urethral strictures up to 2 centimeters were treated utilizing anastomotic urethroplasty. The stricture section was removed after the urethral mobilization, and a typical end-to-end anastomosis was carried out. The 14Fr silicon catheter was used for all urethroplasty surgeries. The perineum was then closed in layers after a suprapubic catheter and drain were placed in certain cases.

Technique of progressive perineal urethroplasty

Patients with pelvic fracture urethral injury (PFUI) would undergo PPU three months after trauma. Length of stricture was assessed with the help of RGU and VCUG films. The perineum was incised in an inverted Y form while under general anesthesia. The urethra was dissected circumferentially, up to the penoscrotal junction in the distance and proximally up to the scarred fibrotic tissue. A flexible cystoscope was used to locate the proximal end of the ill-fibrotic urethra. After the fibrotic and stenosed segment was removed, an end-to-end urethral anastomosis was done. To accomplish a tension-free anastomosis, lengthier strictures may require inferior pubectomy and crural separation. All urethroplasties were performed with an adequately sized silicone perurethral catheter in place, along with suprapubic catheter insertion.

Follow-up

Three weeks following the date of surgery, a pericatheter urethrogram was performed, and the perurethral catheter was removed. Four to five days later, the suprapubic catheter was removed. Sexual activity was permitted for patients two months after the catheter was removed. The IIEF questionnaire was conducted again at three, six, nine, and 12 months following surgery. To rule out arterial insufficiency, venous leak, or mixed etiology as the vascular cause of ED, patients having an initial preoperative IIEF-5 rating of less than 22 and those with a score of less than 22 that emerged after surgery received repeat pharmacological CDUS at six months.

Statistical analysis

The statistical analysis has been done using SPSS program for Windows, Version 17.0 (SPSS, Chicago, Illinois). Categorical variables have been displayed as percentages as well as absolute numbers, whereas continuous data are demonstrated as mean ± SD. The data's normality was checked before statistical analysis. Fisher's exact test or chi-square test has been used to assess categorical variables. Bonferroni's post hoc testing was used after repeated measures analysis of variance (ANOVA) to evaluate continuous variables over time within the groups. Results before and after surgery have been compared by utilizing a paired t-test. A significance criterion of a p-value less than 0.05 was established.

## Results

Fifty-eight patients met the inclusion criteria; eight of them underwent PPU for PFUI, and 50 of them underwent urethroplasty for AUS (either in the penile or bulbar region). The average age of all patients with stricture was 37.74 ± 8.55, and the largest proportion of patients were between the ages of 31 and 40 (Table 1). Patients were classified, as per the site of urethral stricture, into three groups: bulbar, penile, and bulbomembranous groups. Strictures were seen in the bulbar urethra in 52% of patients, the penile urethra in 34%, and the bulbomembranous urethra in 14%. Mean stricture length in our study was 2.27cm ± 0.96 for bulbar strictures, 2.67cm ± 0.66 for penile strictures, and 2.09cm ± 0.83 for strictures in the bulbomembranous region. Idiopathic (40%) causes were the most frequent causes of bulbar and penile urethral stricture. All PFUIs were due to trauma with pelvic fracture (Table 2). Twelve of the 30 patients who had bulbar urethral strictures had end-to-end anastomosis, and 18 had BMG urethroplasty. Every patient who had a penile urethral stricture had a BMG urethroplasty. PPU was performed on all bulbomembranous urethral stricture patients. All patients with penile urethral stricture had the graft placed using either the dorsolateral technique (15 patients) or the dorsal inlay approach (five patients). Seven out of the fourteen patients receiving BMG urethroplasty for bulbar urethral stricture had the graft placed dorsolaterally; the other patients underwent dorsal onlay and dorsal inlay procedures. In three patients graft was placed using the dorsal inlay + ventral onlay technique (Palminteri technique). Mean preoperative Qmax among bulbar urethral stricture patients was 5.43 ± 1.65 mL/sec. While among penile urethral stricture patients, it was 5.10 ± 1.17ml/sec. Preoperative IIEF-5 questionnaire was given to all patients, and the IIEF-5 score was recorded. Among patients with AUS, 30 (60%) of the men reported having some form of ED before surgery (Table 3). Postoperatively, at three months, 33 (66%) men reported with ED, while after 12 months, 28 (56%) men reported with ED.

**Table 1:** Age distribution among patients undergoing urethroplasty This is descriptive statistics showcasing the age group among all patients undergoing urethroplasty.

Age Groups	Frequency	%
21 - 30 yrs	13	22.4%
31 - 40 yrs	22	37.9%
41 - 50 yrs	19	32.8%
>50 yrs	4	6.9%
Total	58	100%
Mean age (SD)	37.74 (8.55)	

**Table 2:** Most common etiology of stricture among all age groups Chi-square test has been used here and signifies a highly significant p-value <0.001. PF: pelvic fracture.

Cause of stricture	Site of stricture			p-value
	Bulbar	Penile	Bulbomembranous	
	Frequency (%)	Frequency (%)	Frequency (%)	
Failed Hypospadias	0	1 (5.0%)	0 (0.0%)	<0.001
Idiopathic	12 (40%)	8 (40.0%)	0 (0.0%)	
Inflammatory	7 (23.3%)	5 (25.0%)	0 (0.0%)	
Iatrogenic	11 (36.6%)	6 (30.0%)	0 (0.0%)	
Traumatic With PF	0 (0.0%)	0 (0.0%)	8 (75.0%)	
Total	30 (100%)	20 (100%)	8 (100%)	

**Table 3:** Overall prevalence of erectile dysfunction among different groups of patients This is descriptive statistics showcasing the prevalence of ED in different groups. IIEF: International Index of Erectile Function.

	Mild to moderate ED	Mild ED	No ED
Bulbar			
Pre op IIEF	2 (6.7%)	14 (46.7%)	14 (46.7%)
Post op IIEF_3 months	6 (20%)	13 (43.3%)	11 (36.7%)
6 months	4 (13.3%)	13 (43.3%)	13 (43.3%)
9 months	4 (13.3%)	12 (40%)	14 (46.7%)
12 months	2 (6.7%)	13 (43.3%)	15 (50%)
Penile			
Pre op IIEF	3 (15%)	11 (55%)	6 (30%)
Post op IIEF_3 months	3 (15%)	11 (55%)	6 (30%)
6 months		13 (65%)	7 (35%)
9 months		13 (65%)	7 (35%)
12 months		13 (65%)	7 (35%)
Bulbomembranous			
Pre op IIEF	2 (25%)	3 (37.5%)	3 (37.5%)
Post op IIEF_3 months	4 (50%)	4 (50%)	
6 months	4 (50%)	4 (50%)	
9 months	4 (50%)	4 (50%)	
12 months	2 (25%)	5 (62.5%)	1 (12.5%)

The mean preoperative IIEF score for patients with bulbar urethral stricture was 20.70 ± 2.69, and 53.3% (16/30) experienced preoperative ED. At three months, 63.3% (19/30) of the patients had postoperative ED. 12 (40%) patients had worsening of IIEF scores, out of which eight patients had preoperative erectile dysfunction. New-onset ED was noted in four patients. Eleven patients (36.6%) had no change in IIEF scores, while seven patients (23.3%) had improved scores after surgery. After three months, the mean IIEF score did not statistically significantly decrease (p-value 0.061) (Table 4). About 11 out of 12 patients (91.6%) with worsening of IIEF scores recovered their erectile function to preoperative status. Additionally, there wasn’t any significant difference in IIEF ratings at the 12-month follow-up (p-value 1.000). The erectile function of all four individuals with newly diagnosed ED returned to normal. Erectile function significantly improved over 12 months (p-value 0.007), and there were 15 (50%) ED patients overall (Table 3).

**Table 4:** Comparison of mean IIEF score among patients with bulbar urethral stricture undergoing urethroplasty One-way ANOVA with post hoc Tukey test has been used here for analysis. IIEF: International Index of Erectile Function.

IIEF		Mean Difference	Std. Error	p-value	95% Confidence Interval for Difference	
					Lower Bound	Upper Bound
Pre op IIEF	Post op IIEF 3 months	0.9	0.305	0.061	-0.025	1.825
	6 months	0.333	0.337	1.000	-0.69	1.356
	9 months	0.1	0.308	1.000	-0.837	1.037
	12 months	-0.033	0.29	1.000	-0.913	0.846
Post op IIEF 3 months	6 months	-.567	0.133	0.002	-0.97	-0.163
	9 months	-.800	0.155	<0.001	-1.27	-0.33
	12 months	-.933	0.166	<0.001	-1.437	-0.43
6 months	9 months	-0.233	0.079	0.059	-0.472	0.005
	12 months	-.367	0.112	0.028	-0.708	-0.026
9 months	12 months	-0.133	0.063	0.434	-0.325	0.058

Patients who received EEAU had a more noticeable decline in erectile function after surgery, though most of them recovered, and their IIEF score did not change significantly after a year, according to a comparison of the end-to-end anastomotic and BMG urethroplasty subgroups.

Preoperatively, 70% (14/20) of individuals with penile urethral stricture had ED, and the mean preoperative IIEF score was 19.75 ± 3.14. A total of 70% (14/20) of patients experienced ED after surgery at three months. Four (20%) patients had worsening of IIEF scores, out of which three patients had preoperative erectile dysfunction. It was discovered that one patient had a newly developed ED. The scores of eight patients remained unchanged. Eight patients had improved IIEF scores after surgery. However, these changes in IIEF scores were shown to be statistically insignificant (p-value 1.000). After 12 months, three out of four patients (75%) with worsened scores recover to preoperative status. One patient with a new onset of ED also recovers his erectile function. After a year of follow-up, patients' erectile function significantly improved, and there has been no statistically significant shift in their IIEF ratings (p-value 0.467) (Tables 3, 5).

**Table 5:** Comparison of mean IIEF score among patients undergoing urethroplasty for penile urethral stricture One-way ANOVA with post hoc Tukey test has been used here. IIEF: International Index of Erectile Function.

IIEF		Mean Difference	Std. Error	p-value	95% Confidence Interval for Difference	
					Lower Bound	Upper Bound
Pre op IIEF	Post op IIEF 3 months	-0.05	0.4	1.000	-1.321	1.221
	6 months	-0.8	0.484	1.000	-2.338	0.738
	9 months	-0.95	0.473	0.589	-2.451	0.551
	12 months	-1	0.47	0.467	-2.492	0.492
Post op IIEF 3 months	6 months	-.750	0.16	0.002	-1.258	-0.242
	9 months	-.900	0.161	<0.001	-1.41	-0.39
	12 months	-.950	0.153	<0.001	-1.437	-0.463
6 months	9 months	-0.15	0.082	0.828	-0.41	0.11
	12 months	-0.2	0.092	0.421	-0.491	0.091
9 months	12 months	-0.05	0.05	1.000	-0.209	0.109

Patients with penile and bulbar urethral stricture did not significantly differ in their mean postoperative IIEF scores at three months (p-value 1.000) (Table 6). However, erectile dysfunction was observed in a greater proportion of individuals with bulbar urethral stricture (40%) than those with penile urethral stricture (20%). The technique of urethroplasty did not affect the overall outcome of surgery about IIEF scores. Patients who received BMG urethroplasty recovered their erectile function similarly to those who received end-to-end anastomotic urethroplasty. Graft placement methods such as dorsal onlay, dorsal inlay, Palminteri technique, and dorso-lateral graft placement were employed. None of the patients had a significant decrease in IIEF scores after 12 months, irrespective of the technique used.

**Table 6:** Comparison of general trend of mean IIEF-5 score among different groups of patients undergoing urethroplasty One-way ANOVA with post hoc Tukey test has been used here. IIEF: International Index of Erectile Function.

IIEF	Groups			p-value	Bulbar V/S Penile	Bulbar V/S Bulbomembranous	Penile V/S Bulbomembranous
	Bulbar	Penile	Bulbomembranous				
Pre op	20.7 ± 2.69	19.75 ± 3.14	19.12 ± 3.04	0.301	0.497	0.366	0.864
Post op IIEF 3 months	19.8 ± 3.04	19.8 ± 2.67	16.75 ± 2.82	0.027	1.000	0.028	0.038
6 months	20.37 ± 3.03	20.55 ± 2.35	17.5 ± 2.62	0.025	0.971	0.031	0.029
9 months	20.6 ± 2.87	20.7 ± 2.43	18.25 ± 2.96	0.081	0.991	0.088	0.092
12 months	20.73 ± 2.75	20.75 ± 2.4	18.5 ± 2.93	0.096	1.000	0.097	0.117

Among all patients with PFUI mean preoperative IIEF score was 19.12 ± 3.04, and the preoperative prevalence of ED in PFUI was 62.5% (5/8). After three months of follow-up, there was a worsening of the IIEF score in all eight patients (100%). It was discovered that three patients had newly developed ED. After three months, the mean IIEF score significantly decreased to 16.75 ± 2.82 (p-value 0.002), and after six months, the mean IIEF score significantly changed again, reaching 17.50 ± 2.62 (p-value 0.005). But at nine months, there is a significant improvement in mean IIEF scores (p-value 0.025) as compared to the mean score at six months, and this improvement is also maintained at 12 months. Overall, there wasn’t any significant change in the IIEF score after 12 months (p-value = 0.112), and one patient experienced a new onset of erectile dysfunction. Five patients restored their erectile function to the preoperative condition after 12 months (Table 7).

**Table 7:** Comparison of mean IIEF score among patients with pelvic fracture urethral injury (bulbomembranous urethra) before and after urethroplasty One-way ANOVA with post hoc Tukey test has been used here. IIEF: International Index of Erectile Function.

IIEF		Mean Difference	Std. Error	p-value	95% Confidence Interval for Difference	
					Lower Bound	Upper Bound
Pre op IIEF	Post op IIEF 3 months	2.375	0.324	0.002	1.07	3.68
	6 months	1.625	0.263	0.005	0.565	2.685
	9 months	0.875	0.227	0.062	-0.038	1.788
	12 months	0.625	0.183	0.112	-0.112	1.362
Post op IIEF 3 months	6 months	-0.75	0.313	0.479	-2.013	0.513
	9 months	-1.5	0.378	0.054	-3.023	0.023
	12 months	-1.750	0.366	0.020	-3.225	-0.275
6 months	9 months	-.750	0.164	0.025	-1.409	-0.091
	12 months	-1.000	0.189	0.011	-1.761	-0.239
9 months	12 months	-0.25	0.164	1.000	-0.909	0.409

**Figure 1:**
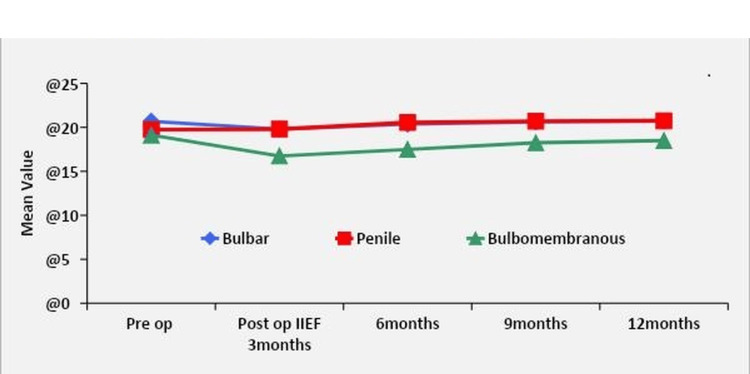
Comparison of overall trend of change in IIEF score in all patient groups undergoing urethroplasty

Most individuals who underwent urethroplasty for AUS and experienced de novo erectile dysfunction saw a gradual improvement in their erectile function; most patients regained baseline erectile function five to twelve months after therapy (Figure 1).

On CDUS, there was no significant shift in mean PSV and EDV values when the preoperative group was compared to the postoperative group among patients with bulbar urethral stricture (p-value 0.034 and 0.0572, respectively). All patients had RI >0.9 (Table 8). Similarly, no significant change in mean PSV and EDV was noted in patients with penile urethral stricture (p-value 0.752 and 0.263, respectively). All patients had RI >0.9 (Table 9). Among all patients with PFUI (bulbomembranous), no significant change was noted in preoperative and postoperative PSV and EDV values (p-value 0.621), with all patients having RI>0.9 (Table 10). These results demonstrated that the postoperative results of PSV, EDV, and RI were unaffected by free graft urethroplasty or anastomotic urethroplasty.

**Table 8:** Comparison of mean PSV and EDV in doppler study of bulbar urethral stricture patients before and after undergoing urethroplasty A paired t-test has been used and shows a significant p-value of <0.05. PSV: peak systolic velocity; EDV: end diastolic velocity; SD: standard deviation.

		N	Mean ± SD	Mean Difference ± SD	p-value
PSV>25cm/sec	Preoperative Doppler	16	44.44 ± 2.10	0.56 ± 0.96	0.034
	Postoperative Doppler	16	43.88 ± 1.96		
EDV<5cm/sec	Preoperative Doppler	16	3.20 ± 0.73	(-)0.05 ± 0.35	0.572
	Postoperative Doppler	16	3.25 ± 0.58		

**Table 9:** Comparison of mean PSV and EDV in Penile Doppler study of penile urethral stricture patients before and after undergoing urethroplasty A paired t-test has been used here. PSV: peak systolic velocity; EDV: end diastolic velocity; SD: standard deviation.

		N	Mean ± SD	Mean Difference ± SD	p-value
PSV>25cm/sec	Preoperative Doppler	14	41.50 ± 2.62	(-)0.07 ± 0.83	0.752
	Postoperative Doppler	14	41.57 ± 2.41		
EDV<5cm/sec	Preoperative Doppler	14	3.43 ± 0.55	(-)0.14 ± 0.46	0.263
	Postoperative Doppler	14	3.57 ± 0.65		

**Table 10:** Mean PSV and EDV in Doppler study of PFUI (bulbomembranous) patients before and after undergoing urethroplasty A paired t-test has been used here. PSV: peak systolic velocity; EDV: end diastolic velocity; SD: standard deviation.

		N	Mean ± SD	Mean Difference ± SD	p-value
PSV>25cm/sec	Preoperative Doppler	5	29.20 ± 1.30	0.20 ± 0.84	0.621
	Postoperative Doppler	5	29.00 ± 1.00		
EDV<5cm/sec	Preoperative Doppler	5	4.00 ± 0.35	(-)1.00 ± 0.42	0.621
	Postoperative Doppler	5	4.10 ± 0.22		

## Discussion

Genital sensitivity problems, penile curvature or chordee, ejaculatory dysfunction, and erectile dysfunction are all included in the general phrase sexual dysfunction. Current research indicates that de novo sexual dysfunction following urethroplasty is rare. It is reported to be around one percent (0-38 percent) after Anterior urethroplasty and around three percent (0-34 percent ) after PFUI repair [2,5].

De novo erectile dysfunction post-urethroplasty may arise from injury to the artery of the bulbar urethra or to vascular connections among spongiosa and cavernosa during movement of the bulbar urethra or from damage to cavernous nerves during intercrural dissection of the urethra (cavernous nerves run at one and eleven o’clock positions approximately 3 millimetres outside the spongiosa) [6-8].

Mundy [9] was the first urologist who stated the incidence of erectile dysfunction after urethroplasty in 1993. Of 200 patients in their study, 114 had graft urethroplasty and 86 had anastomotic transperineal and abdominoperineal urethroplasty. After graft urethroplasty, the permanent erectile dysfunction rate was 0.9%, while the rate following abdominoperineal urethroplasty and anastomotic transperineal was five percent. However, preoperative erectile function was not evaluated in this study. These findings led to further evaluation of erectile dysfunction and other forms of sexual dysfunctions like diminished glans coolness or turgidity, chordee, ejaculatory dysfunction, etc. Following urethroplasty by reconstructive urologists. Initially, it was reported that erectile dysfunction post-urethroplasty was maximum in elderly patients having longer strictures (6.8 centimetres versus 4.4 centimetres) [10]. Newer research denied this theory, and a recently conducted meta-analysis found no correlation between stricture length and incidence of postoperative erectile dysfunction [2,7].

Theoretically, there is a higher possibility of post-urethroplasty sexual dysfunction and a higher danger of damage to arteries and cavernosal nerves following bulbar urethroplasty. Some authors have reported that erectile and ejaculatory dysfunction is more frequent after urethroplasty for bulbar urethral stricture [11]. No evidence is there linking the penile urethral stricture length or type of graft utilized to the prevalence of sexual dysfunction following surgery. In their study, Erickson et al. assessed sexual dysfunction after graft urethroplasty for penile urethral stricture and found no significant decrease [1].

Xie et al. [12] assessed sexual dysfunction post-urethroplasty for pan-anterior USD (>10 cm) and reported penile shortening in six patients, transient penile edema in five individuals, and ED in 25% of individuals at six months post-urethroplasty. If there was no intercrural dissection during urethroplasty or if the dissection did not reach into the proximal bulbar urethra, they concluded that there was a low prevalence of sexual dysfunction.

Different studies have evaluated whether non-transecting techniques of urethroplasty are superior to transecting techniques concerning sexual dysfunction. The rates of erectile and ejaculatory dysfunction were similar between the two techniques [13,14].

We showed that, in comparison to anterior (bulbar and penile urethra) urethral reconstruction, the IIEF-5 scores dropped more dramatically following PFUI. In patients with PFUI, the surgeon finds it more challenging to mobilize the corpus spongiosum. After all, they usually have more scar tissue. Therefore, the likelihood of ED is increased if neurovascular bundles are damaged during surgery. These conclusions with respect to the previous study by Xie et al. [15] for evaluation of erectile function in both anterior and posterior urethral strictures.

Erectile function gradually improved for the majority of patients who had urethroplasty for AUS and who developed de novo erectile dysfunction; most patients returned to baseline erectile function between five and twelve months following therapy (Figure 1). Similar recovery in erectile function has been reported in previous studies by Erickson et al. [1], Dogra et al. [7], and Singh et al. [16] in their study of patients with AUS. This implies that both short-term and long-term consequences could be responsible for erectile dysfunction following urethroplasty.

CDUS has been used in some previous studies to evaluate vasculogenic causes of ED. Mondal et al. [17] for AUS did not show any significant change in IIEF scores and PSV and RI in penile Doppler done six months after surgery. Hosseini et al. [18] for evaluation of erectile function after PPU did not show any significant change in PSV six months after surgery. In these patients, the involvement of arteriogenic (bulbar artery), neurogenic (perineal nerve), and psychological aspects is not well known.

In our study, psychological impact and associated factors were not evaluated. Erectile function is typically greatly impacted by the morbidity of this disease itself. Since evaluating the disease's natural progression was our goal, the function of phosphodiesterase-5 inhibitors was not examined. We were unable to do CDUS on all patients before surgery due to financial constraints.

Limitations

Despite having a sizeable patient population, this study's subgroup numbers are small, which could have an adverse effect on the findings. Specific assessments for neurogenic and psychogenic causes of erectile dysfunction were not done. Surgery was performed by multiple surgeons, which may lead to bias.

## Conclusions

After this study, we conclude that patients with PFUI and AUS have a significant prevalence of ED. Erectile function may temporarily deteriorate after urethroplasty, although most patients return to their preoperative state within a year. The rate of new-onset ED is low in both AUS and PFUI patients. After these procedures, pharmacological CDUS does not significantly show the vascular events that cause ED. Either a neurogenic or a psychogenic pre-existing condition may be the root cause of ED. Psychometric analysis may be included in future research.

## Figures and Tables

**Figure 2:**
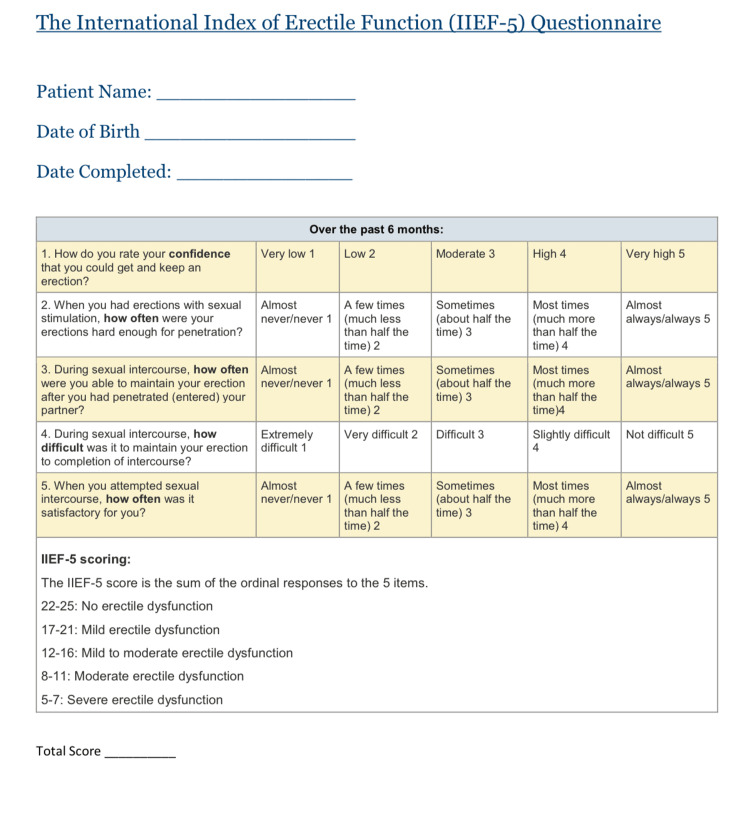
International Index of Erectile Function – 5 item version (IIEF-5) questionnaire to assess presence and severity of erectile dysfunction An open-access questionnaire has been previously employed by Erickson et al. in their prospective analysis of erectile dysfunction following anterior urethroplasty.

## Appendices

Appendix 1 
